# Equity, justice, and social values in priority setting: a qualitative study of resource allocation criteria for global donor organizations working in low-income countries

**DOI:** 10.1186/s12939-021-01565-5

**Published:** 2022-02-08

**Authors:** Lydia Kapiriri, S. Donya Razavi

**Affiliations:** Department of Health, Aging and Society, McMaster University, 1280 Main street West, Hamilton, Ontario Canada

**Keywords:** Equity, Social justice, Social values, Development assistance partners (DAP), Health systems, Low-income countries, Priority setting, Resource allocation

## Abstract

**Background:**

There is increasing acceptance of the importance of social values such as equity and fairness in health care priority setting (PS). However, equity is difficult to define: the term means different things to different people, and the ways it is understood in theory often may not align with how it is operationalized. There is limited literature on how development assistance partner organizations (DAP) conceptualize and operationalize equity in their health care prioritization decisions that affect low-income countries (LIC). This paper explores whether and how equity is a consideration in DAP priority setting processes.

**Methods:**

This was a qualitative study involving 38 in-depth interviews with DAPs involved in health-system PS for LICs and a review of their respective webpages.

**Results:**

While several PS criteria were identified, direct articulation of equity as an explicit criterion was lacking. However, the criterion was implied in some of the responses in terms of prioritizing vulnerable populations. Where mentioned, respondents discussed the difficulties of operationalizing equity as a PS criterion since vulnerability is associated with several varying and competing factors including gender, age, geography, and income. Some respondents also suggested that equity could be operationalized in terms of an organization not supporting the pre-existing *inequities*.

Although several organizations’ webpages identify addressing inequities as a guiding principle, there were variations in how they spoke about its operationalization. While intersectionalities in vulnerabilities complicate its operationalization, if organizations explicitly articulate their equity focus the other organizations who also have equity as a guiding principle may, instead of focusing on the same aspect, concentrate on other dimensions of vulnerability. That way, all organizations will contribute to achieving equity in all the relevant dimensions.

**Conclusions:**

Since most development organizations support some form of equity, this paper highlights a need for an internationally recognized framework that recognizes the intersectionalities of vulnerability, for mainstreaming and operationalizing equity in DAP priority setting and resource allocation. Such a framework will support consistency in the conceptualization of and operationalization of equity in global health programs. There is a need for studies which to assess the degree to which equity is actually integrated in these programs.

## Research highlights

Equity has become an increasingly important focus in the health and social science literature, however, equity is a contested concept.

While development assistance partners supporting health development subscribe to equity as a guiding principle, they struggle with its operationalization.

There is need for a general framework that explicitly conceptualizes the operationalization of equity in health development.

## Introduction

Development Assistance Partners (DAPs) or donors have significant influence on health care and health-systems priority setting (PS) in low-income countries (LIC) [1–4]. DAPs are relied upon as they have the resources needed to enact priorities, and because their experience and related knowledge means that they are well-equipped to determine what those priorities should be. DAPs are often tasked with determining the most efficient, effective, and equitable ways to provide aid, since the resources can be scarce, and needs can be great [5]. To navigate these complex circumstances, DAPs often make use of established PS criteria.

There is a range of criteria from which DAPs can choose in establishing priorities, for example those identified in some of the priority-setting frameworks [6–9]. Among the many criteria, equity has emerged as an important criterion, which is often not considered in many prioritization processes [5, 7, 10]. Within the scholarly literature, equity has several definitions and conceptualizations. The economics literature tends to frame equity in terms of *inequity* rather than equity [11, 12], and often juxtaposes it with the concept of efficiency [13]. While the aim of efficiency is to reduce wastefulness, equity is focused on fairness both process and ultimate distribution of resources. Both the political sciences and philosophy literature defines equity in terms of [social] justice and fairness [14–16]. The health literature often defines equity as the absence of systematic disparities in health and its determinants [17–19]. The literature on health-system priority setting draws on these various definitions of equity when determining health priorities and the resulting resource allocation decisions. In the field of priority setting, equity is often conceptualized in terms of fairness in the distribution of benefits and burdens in society, in particular the fair and just distribution of scarce health resources [6, 20–25]. This literature further discusses equity in terms of horizontal and vertical equity. According to the principle of horizontal equity, people with the same health needs should have the same/ similar access to health care. Conversely, vertical equity supports the notion that people with different (unequal) health needs should have unequal access to health care [8, 23, 26–28]. The equity criterion can be operationalized in either in terms of representation and participation in the prioritization process; or as criterion that explicitly guides the decision making process; whereby different vulnerabilities (such as age, gender, economic status, residence…) are identified and considered in priority setting and resource allocation. Equity in participation supports the representation of the vulnerable populations in decision making [27, 29–32]). However, equity in participation may not necessarily result in equitable resource allocation. Achieving equity in the outcome of priority setting processes requires the consideration of equity as criterion [33, 34].

The concept of equity has different meanings for different people (as discussed above) and when used in daily interactions. What counts as just or fair to one person or group looks inequitable to another. Does equity mean fairness across ages, genders, geographical regions, or social classes? What about individuals that are disadvantaged in more than one way? The wide range of meanings afforded to the concept of equity—and the challenges posed with balancing these meanings—is one of the elements this paper explores.

In addition, this paper also looks at the gap that exists between what is valued in theory and what happens in practice. After all, DAPs must set priorities that not only equitably meet the needs of the population they are serving, but they must also meet a number of other less idealistic, more practical criteria. For example, alongside equity, DAPs must pay close attention to the financial sustainability of their recommendations and projects. Indeed, research has shown that when resources are limited and not all interventions can be pursued, it can be difficult to meet a vague and often multifaceted definition of equity [35].

There is little literature exploring the complicated process of achieving equity; there is also a gap in the scholarship when it comes to understanding how the concept of equity is applied to setting priorities for low-income countries. This paper rectifies these gaps by exploring how global DAPs conceptualize and operationalize equity as a criterion for priority setting.

This paper had two objectives. The first objective was to describe the criteria used by global-level health DAPs in their prioritization process, with a focus on identifying and understanding criteria related to equity. The second objective was to determine the importance and emphasis the respondents placed on equity and how equity is operationalized when setting LIC health priorities within their organizations.

## Materials and methods

This was a qualitative study involving global level interviews with DAPs, which were conducted between 2015 and 2017. It also included a review of related information about PS and equity obtained from the DAPs’ websites.

### Data collection and analysis

#### Study sample and sampling strategy

We shared the study information with the largest DAP organizations that support LIC health systems. We accessed their contacts through their respective webpages. The initial contacts were program directors of either the HIV/AIDs, NCD, MNCH, Emergencies, Vaccines or health systems programs within their organization. Once these were interviewed, we requested them to share our email with others that they deemed suitable to respond to our questions. These contacted us and were interviewed. We stopped requesting for additional contacts once we achieved saturation.

The interviews were conducted via skype and telephone by the PI and a trained research assistant. A pilot-tested interview guide was used. The interview guide included general questions about priority setting, the criteria used in priority setting, with specific focus on equity and its role. Interviews lasted between 45 and 60 min. All interviews were audio recorded with permission from respondents and were transcribed verbatim.

NVIVO-10 was used in analysing the transcribed data. Three members of the research team coded one interview. After the independent coding, the team members met and discussed the code names each had identified from the interview. Codes that were consistent were adopted for use; codes where there was no agreement were discussed and an agreed-upon code identified. The edited list of codes was then used for coding the rest of the interviews. However, an open stance was maintained to identify any emerging codes, which were discussed and added to the code list.

This paper reports the findings that arose from synthesizing the data related to criteria for priority setting codes, and specifically, equity. The initial coding identified several responses, which were labelled as “criteria”. At an abstract level, systematic review of all the identified criteria revealed a pattern whereby related criteria were grouped under more broad categories e.g. vulnerability/equity criteria, which are presented in the results section. We also explicated respondents’ understanding/conceptualization of the various criteria, how they spoke about the operationalization of the concept in their programming, and the challenges associated with this operationalization.

The organizations’ webpages were reviewed to assess the degree to which equity is identified either as a guiding principle, or criterion. If identified, we sought to understand how, if at all, they talk about how they operationalize equity in health program implementation in low income countries. This information complemented the interview data.

### Ethics

This study was reviewed by and received ethics clearance from McMaster Research Ethics Board.

## Results

We conducted a total of thirty-eight in-depth interviews with DAP stakeholders from seven organizations that support LIC health systems. In reporting respondents are identified according to their organizations (corresponding respondents’ IDs): UN agencies (9) (UN_), public-private partnerships and alliances (7) (PPP_), NGOs (8) (NGO_), bilateral organizations (4) (BL_), foundations (4) (F_), national health research agencies (3) (NHR_), and financial institutions (3) (FIN_).

The interviews revealed that there was an overall agreement among the respondents that equity is an important consideration when setting health system priorities for low-income countries. However, they also identified challenges when trying to define and operationalize the concept of equity. To provide context for the discussion on equity, the first section describes the PS criteria as identified by the respondents. This is followed by an in-depth look at the stakeholders’ understanding of equity, and the place equity is afforded within the PS process. Throughout, there is a focus on what the respondents identified as the strengths provided and the problems posed by using equity as a criterion in priority setting.

### Criteria used for PS

Respondents were asked to list the criteria that they use when setting health system priorities for low income countries. Across all the organizations, nine groups of common criteria emerged in interviewees’ responses. These included economic considerations, evidence, expertise, feasibility, impact, innovation, interests, sustainability, and vulnerability (see Table 1 for a division of criteria by type of organization).

**Table 1:** Priority setting Criteria identified by DAP respondents

		Type of organization						
Criteria	Explanation	Bilateral organization	Financial organization	Foundations	National health research agencies	NGOs	Public-private partnership and alliances	UN agencies
Economic considerations	Including economic evaluations, elements of supply-demand, and financial resources.	Cost-effectiveness	Cost-effectiveness	Cost-effectiveness	–	Cost-effectiveness, maximizing benefit, funding	Market considerations & cost per death averted	–
Evidence	Use of evidence in decision-making	X	–	X	X	X	X	X
Expertise	Knowledge & skills, experience, and comparative advantage	–	–	Comparative advantage & filling a gap	Capacity [for research]	Comparative advantage & filling a gap	X	–
Feasibility	Whether the organization has the capacity and resources to carry out the intervention(s)	–	–	X	X	X	X	–
Impact	Including the health impact on burden of disease, mortality and morbidity, and poverty	Overall impact	Impact on poverty & impact on mortality	Overall impact & burden of disease	–	Burden of disease	Health impact, impact on mortality and morbidity & burden of disease	Impact on mortality
Innovation	Original and/or creative solutions, tools, or strategies to address health challenges	–	–	X	X	–	–	–
Interests	Political, organization/funder, personal, global priorities and initiatives	Political interests (donor & recipient)	–	Personal & organizational interests	–	Alignment with global Initiatives	–	Alignment with global priorities
Sustainability	Both financial and political sustainability of activities & programs	–	–	–	–	–	X	X
Vulnerability	Disadvantage, (lack of) inclusiveness, (un)equal access or distribution of resources, disparities in BOD	–	–	–	–	X	X	X

Almost all the respondents identified criteria such as cost-effectiveness, evidence-based interventions, and impact. However, none of the respondents explicitly cited equity among their core criteria. This does not mean that the concept of equity was entirely absent, as respondents mentioned ideas related to equity and ideas that implied its presence. For example, some respondents referred to the importance of making sure all stakeholders’ views are equitably represented. Others mentioned the value of identifying and responding to vulnerability. Addressing vulnerability implies acknowledging those who stand to benefit most from equity-based interventions. From this, we can say that although the concept of equity was not explicitly identified by the respondents as one of their criteria, it was implied in many of the criteria. For these respondents, the idea of equity was an inherent principle contained within other PS criteria—an idea that is explored further below.

However, the webpage review revealed that many of the organizations represented among our study sample mandate equity as part of their policies. These identify equity as a core value and an integral part of their organizational mission, as well as a goal of their programming. Interestingly, review of the documented criteria revealed a pattern that is consistent with the interviews; equity is not always listed as an explicit criterion. The organizations also tend to operationalize the equity criterion in terms of vulnerability e.g. targeting vulnerable or marginalized populations (based on gender, geography, age, and immigration/refugee status).

The fact that equity did not come up when the respondents were asked to list their organizations’ criteria for PS, and that while equity is often a stated core value for DAP, it is not often explicitly listed as a criterion used to make priority setting decisions highlights the gulf between equity as a theoretical principle and its practical application when setting health priorities, another idea explored below.

### Defining equity

When respondents were asked to expand on the concept of equity, they consistently talked about equity in terms of concern for vulnerable and marginalized populations. However, because respondents defined equity by referring to examples where it featured in their work, no single, shared vulnerable and marginalized population emerged. For example, some respondents identified equity as a concern with respect to geography (residence), others with respect to gender, and still others with respect to socioeconomic gaps as exemplified in the quotes below.

#### Geography

*Many aid agencies … will set their operations in and around the cities because of logistical conveniences, whereas those most in need could be ten hours away. And this is where we would be negotiating and arguing and advocating and trying to reach those actually truly most in need.* (NGO_2)

#### Gender

*Gender equality has always been … a major policy priority for the development cooperation, and linking gender equality with sexual health and reproductive life is also a part … If you have a strong gender equality perspective … that completely explains why sexual and reproductive health arrives as a priority.* (UN_3)

#### Socioeconomic factors

*On the equity issue, we started to look at some countries like for example Ethiopia or China, or we found out that it was needed to focus on the … more vulnerable and poorest quintile of these countries.* (NGO_8)

#### Additional vulnerable populations

*Are these services … being delivered in a non-stigmatizing way, in a non-discriminatory way? And are they really getting to the right people? Are they getting to the ones who are the most vulnerable? Sex workers, for example.* (UN_1)

Equity can mean balancing the priorities to account for gender (women), geography (hard to reach areas or rural dwellings), age (children and the elderly), and income (poor), among other factors—or possibly for more than one of these factors at once. Respondents noted the difficulties in trying to effectively cater for all possible vulnerabilities as a single organization. This results in organizations focusing on those vulnerabilities that are associated with their mandate e.g. organizations supporting maternal and child health programs perceiving equity in terms of gender and age.

### Defining equity in terms of inequity

While the study focus was on the equity, some of the respondents framed equity in relation to its inverse: inequity. As one respondent explained:

*Inequities have been a big thread … We have had publication looking at why investing in those most at need or those most disadvantaged is not just a moral imperative … but it's also cost-effective, and it’s also a good way for countries to look at their universalization of health services and so forth.* (UN_8)

According to this respondent, identified inequities (rather than a more general, ideological pursuit of equity for its own sake) are a catalyst for change. Furthermore, for the above respondent, eradicating inequity is worthwhile not only because it has intrinsic moral/social value, but also because it brings about associated benefits, such as cost-effectiveness and universalization of programs. Helping those most in need can have knock-on benefits. For example, addressing family planning (prioritizing women, often marginalized) can have subsequent positive effects for children (also a marginalized group):

*I think there was a recognition … that there was a knock-on effect with family planning that effected every part of that continuum of care all the way down the line, and which had a lot to do with economical and developmental prospects of countries and regions. If you aren’t able to reduce fertility in certain countries, it's going to be very difficult to achieve other development goals.* (PPP_1)

As for determining what counted as inequities, while most respondents referred to gaps within a country (socioeconomic, geographic, and so on), one respondent pointed out that priorities can be determined by identifying the inequities that separate those in high-income countries (HIC) from those in LICs:

*Should it be the case that in Africa people don’t get antiretroviral drugs just because they are poor, whereas in America because they’re rich they do get it? So, people say, think that’s unfair and therefore you don’t get resources. Therefore, you do try to fund antiretroviral drugs in Africa.* (NHR_2)

Comparing across countries offers a way of identifying areas where there may be resources or structures that can be implemented to the benefit of a marginalized group. In the example above, the respondent identified antiretroviral drugs, which are available in America. Were the drugs to be similarly provided in Africa, an equity imbalance would be addressed.

### Inherent equity

Related to the idea of identifying inequities was the belief—common among respondents—that addressing inequity was at the core of their work, which therefore made it redundant to list equity as a PS criterion. Respondents pointed out that the very nature of their work was to fill gaps in the LICs; this means that they saw themselves as implicitly always working towards equity. To claim equity as a single priority among many was misinterpreting their mission.

However, even among these respondents, there was variability in their understanding of what constituted inherent equity. Some respondents defined the concept of *filling a gap* as simply meeting the needs of the LIC population where they were working; other respondents saw it as seeking out opportunities that other groups working in the same country were not pursuing. As one respondent from a global foundation explained:

*We try to fill in those gaps rather than trying to do the same thing that other funders are doing. I think all funders like to do that; they like to sort of complement and not overlap.* (F_1)

Working in neglected areas does not simply mean finding the most inequitably served groups and assisting them. For some respondents, equity was inherent in their programming when they seek to address areas/issues that are “marginalized” or ignored by the other organizations:

*We tried to identify what can be added value, because there are so many other parties working on these issues. We tried to identify what can be the specific role we can play, … the added value we can bring … and also the gaps … nobody else is working on.* (UN__9)

*As a private foundation … we’re in a position to be able to take more risks and fund some riskier things, which other partners or governments might not be in a position to do.* (F_3)

As these three respondents explained, DAPs often seek opportunities based not only on the needs of the community but also on the strengths of the DAP and the existing gaps. This can mean that DAPs are able to focus on areas that are otherwise overlooked or underserved. However, it can equally mean that there are some groups for which there are no adequately equipped DAPs, which therefore leaves the affected groups further marginalized.

### Contingent equity

Notwithstanding the inherent draw of equity and its implicit place in many of the stakeholders’ missions, some respondents still saw its value as secondary or contingent. For example, with respect to the above mentioned area of expertise, respondents acknowledged that it was most important for them to set priorities that they could meaningfully achieve—even if these were not priorities that were most important from an equity perspective.

In general, respondents saw equity as something worth pursuing only insofar as doing so would allow them to fulfil their organization’s core criteria. Six of the seven groups of respondents interviewed (all but financial organizations) cited evidence as the single most important criterion for the PS process. For these respondents, there was no benefit to undertaking an intervention if it were equitable but lacking in evidence to support its success. An intervention that is equitable in theory is still worthless if it turns out to be ineffective.

In addition to valuing priorities for which there is meaningful evidence, respondents also noted that their stakeholder organizations prioritized interventions that were proven to be sustainable and cost-effective:

*Resources are finite … so, if there’s a more cost-effective vaccine and a less [cost-]effective vaccine, they want to fund the more cost-effective vaccine so the money will go further, and … it’ll have … more overall impact for a given amount of money.* (PPP_1)

While equity may be important, it is still secondary to criteria such as viability, productivity, and value added. When resources are limited, DAPs are under pressure to provide the greatest amount of assistance to the greatest number of people; sometimes, helping those who are the most marginalized is not possible within these constraints.

This struggle to balance evidence and economics with equity was the case even for organizations whose founding principles implied equity. One respondent from a public-private partnership organization summed up this problem by explaining that while their organization’s mission was “*health for everyone*” (a mission that implies equality), their donors still preferred that they pursue programs with the greatest impact for the money contributed. Practical realities outweigh ideological underpinnings.

### Obstacles to equity

#### Competing criteria

Respondents were open about the fact that their struggle to balance concurrent demands—efficacy and economics among them—could lead to biases against equity-based priorities. This was particularly true in situations where other priorities were perceived as more likely to be successful or more likely to reach a greater number of people:

*In the end, we’re looking at how to maximize saving lives … and how do we maximize the limited resources we have so that we can save the maximum amount of lives with the funding that we have partnering with others. That’s the driving point.* (F_2)

#### Feasibility

Similarly, the unfeasibility of an intervention was cited by one respondent as a reason for choosing not to prioritize equity as a criterion:

*We can’t fund research in places where it's not feasible … because they’re conflict zones, or there may not be any sort of infrastructure for … good clinical practice training.* (NHR_1)

Regardless of the equity benefits an intervention might offer, if it is too difficult or too expensive, it may not be pursued. One respondent offered an excellent example of this, explaining how even a viable, productive, cost-effective, evidence-based, equity-driven intervention could be undermined by practical considerations:

*There’s … evidence now to suggest that calcium supplementation when you’re pregnant is important for the outcome … But, you know, clinical studies have shown that the dosage you need to have is three times a day and … from a public health perspective it’s like never going to work*. (BL_1)

In this specific case, there is evidence for the use of calcium supplementation to support a marginalized group (calcium-deprived expectant mothers and unborn babies). However, as the above respondent explains, evidence and equity benefits are not enough to justify an impractical intervention. An intervention may meet all of an organization’s PS criteria, but if it is not feasible, it will still not be pursued.

#### Competing inequities

Respondents were aware of the complexities inherent in applying the concept of equity, and they often cited these complexities in their responses. In particular, respondents noted the difficulty in determining where needs are greatest. Is gender equity more important than serving people in rural areas? Are children more important than those who are the poorest in all age groups?

Across diverse populations with wide-ranging needs, it can be hard to pinpoint who is being served and by what. One respondent explained, “*Most of our programs are set up on the basis of national averages*” (UN_1). Averages may be a necessary simplification when working with national-level populations, but it results in inequities being rendered invisible. Another respondent expanded on this problem:

*Using global or national indicators what we are seeing is … a lot of progress, which is great. If we look beyond the national averages, most of the time we find pockets of population, even in countries like in Latin America where we expect … progress, … the condition of this subset of the population are really as bad as they are in other continent or they were nationwide maybe thirty years ago.* (BL_3)

One way of overcoming these discrepancies is to focus on more granular data. Priorities are often driven by evidence of equity gaps and the need to address various vulnerabilities; looking at more detailed information than national averages helps highlight discrepancies among groups. One respondent explained:

*What do you do about equity? … Most of our health programming … it’s looking for pockets of high mortality but is not necessarily looking inside of those for a particular population, or wealth level, … or vulnerability.* (FIN_3)

For this respondent, focusing on those whose life expectancy is the lowest (regardless of the reason why) is one way to determine who is the neediest among various communities of unequal, unrepresented groups that stand to benefit from a variety of interventions without becoming caught up in the details for why these individuals are most at need. Identifying the greatest in need before choosing the intervention to pursue is a form of detail-oriented, equality-minded PS that focuses not on what is most likely to be successful but rather what is most necessary. This approach resonated with another respondent, who explained:

*The ultimate goal is to is to ensure that people can lead healthy and productive lives and that that the lifesaving interventions that exist are reached, not just people in wealthy developed settings but equally so in other parts of the world. So, it's sort of driven by the mission of all lives have equal value.* (F_3)*.*

### Active pursuit of equity

Of all the respondents interviewed, only one described a fully realized pursuit of equity. This respondent was from an NGO, which was born from observing of the inequitable and unserved needs of people living with disabilities. As the respondent explained,

*Once [people living with disabilities were] … discharged from the hospital, … the amputees had no follow-up care in terms of functional re-adaptation. There was no access at all to prosthetics or walking aids, and it is because of those needs that X came into being.* (NGO_8)

This respondent explained how the organization’s area-specific expertise combined with its identification of an equity gap, to create an inclusive humanitarian response—one that extended outward from the DAP through to other organizations and partners:

*We started thus to train all these humanitarian actors in using better detection methods … in their daily work, because they realized very, very quickly that people with reduced mobility, people with chronic diseases, people with injuries … had special needs that they could not address. And so, we are identified as specialists to deal with this particular problem, but not with the intention that they are just going to refer them to us and then we must be doing the rest. It is rather also again this advocacy work that we do amongst the humanitarian actors … It was also important to work on developing the capability of these humanitarian actors for their response to allow some basic inclusiveness, precisely to give the most appropriate response possible for people with a handicap.* (NGO_8)

This organization was a rare example of equity being not only at the core of their stated mission (as was claimed to be the case by other respondents), but also central to their applied criteria in their day-to-day work.

### Equity’s image problem

There were respondents who suggested that their organizations prioritize equity not for its own sake but rather to avoid a negative association with inequity. In particular, respondents noted that some donors are disinclined to support programs that are seen to be explicitly furthering inequity:

*Of course, X would not be interested in putting millions … of dollars into the country if all that money goes to the well-off people in the capital.* (BL_3)

Even if the motivation to pursue equity is self-interested, the end result is still an equity-based initiative—and therefore a net benefit.

Adding to equity’s image problem is the fact that some funders prefer specific marginalized groups, as do individual DAPs. This can lead to certain groups or areas having a greater proportion of the funding than others (e.g. funding of HIV programs), sometimes in ways that are inequitable or undesirable. Respondents noted that helping one marginalized population can lead to other marginalized populations becoming even further sidelined.

## Discussion

This paper describes the criteria used in PS as identified by the DAP respondents and on their organizations’ webpages. While all the organizations identified equity in their mission statement and goals, most of the respondents did not identify equity as an explicit criterion during the interviews. Respondents tended to talk about equity in other terms related to a concern for vulnerable populations; while others seemed to imply that equity is not perceived as a lone criterion but an inherent value which permeated all their programming. Most of the respondents alluded to equity implicitly when they spoke of considerations such as fairness, social justice and values, equality of access, equally valuing life, inclusion, and vulnerability Consistent with their organizations’ webpage, respondents’ operationalization of equity was associated with identifying vulnerable populations—which varied based on the organization’s expertise and programs of focus.

This study shed light on how DAPs conceptualization of equity may influence active pursuit of equity in their global health programming. The respondents’ conceptualization is consistent with the literature that i) pursuit of equity is often meant to tackle inequalities - the observable differences between subgroups of a population [36], rather pursing the ideal of equity for its own sake; ii) [in]equality, in contrast to [in]equity, is often considered a more concrete, and observable concept [17, 18, 36]. The literature supports the notion that these observable differences can be more easily measured and monitored than equity as a social value or normative principle [17, 18, 36]. The implications of this conceptualization are that DAPs often focus on different dimensions of inequality and specific features of target populations. These dimensions of inequality included gender and age, striving to reach those living in rural and remote regions, and targeting vulnerable populations such as sex workers, injection drug users, and the very poor, when discussing equity as a criterion in their PS process [37].

Whether equity is conceptualized as inherent or contingent in DAPs priority setting processes has implications for its operationalization. DAPs that conceptualized equity as inherent to their core mission may be less concerned with balancing equity with other more immediate or practical criteria such as cost-effectiveness or evidence [17, 18, 38]. As explained by our respondents, this perspective allows DAPs to pursue equity-driven programs and interventions rather than view equity as just another priority setting criteria. Conversely, contingent equity emphasizes the positive externalities and spillover effects of focusing more immediate, practical, and quantifiable priority setting criteria like cost-effectiveness and efficiency [39]. From this perspective, the emphasis is placed on delivering the highest impact interventions, especially in LIC contexts where resources are especially scarce. When a DAP conceptualizes equity as contingent on the other, more tangible criteria, some of the most vulnerable groups may be overlooked and further marginalized.

The challenges identified by the respondents are also consistent with the literature. Competing criteria and the difficulties in measuring equity means that equity often is trumped in favour of other more easily operationalizable PS criteria [8, 10, 40–42]. Notably, criteria like feasibility and efficiency have been found to overrule the equity criterion whereby the operational context of the proposed interventions make them too difficult or cost prohibitive [8, 38] or when it may not be feasibility to identify the most marginalized in each context [43]. Finally, Schneider et al. [44] explain that disadvantaged groups are not uniform and may have competing interests and specialized needs with respect to health system priorities and DAP interventions. It may be difficult for DAPs to determine where needs are greatest since this requires prioritizing among already marginalized groups, which may be inequitable in and of itself.

This paper also highlights the challenges associated with intersectionality [45, 46] when operationalizing equity in low-income countries. Many vulnerable populations embody various, interacting vulnerabilities [47]. Unfortunately, our findings reveal the tendency of organizations to focus on specific and explicit vulnerabilities e.g. women, with the exclusion of other vulnerabilities that these women inevitably embody (such as rural dwelling, poverty, widowhood…) Operationalization of equity as a priority setting criterion cannot prioritize one single dimension of vulnerability to the exclusion of others [46, 47]. The limited focus on a single vulnerability may impact the effectiveness of the piecemeal focused health programs and may either propagate inequity or fail to contribute to promoting equity.

Since the DAPs’ work can either propagate or reduce existing inequalities in health [48, 49], careful consideration of how they can integrate equity in their programs, while leveraging the operations of other organizations to address vulnerabilities that are beyond their scope would support the overall realization of equity among the vulnerable that they seek to serve. This could be conceptualized as a call to both donor harmonization as well as inter-sectoral collaboration at the national level [50, 51]. However, for this to work, there is a need for an overall equity framework that can guide the operationalization of equity at the national level. Furthermore, since equity requires the identification of vulnerable populations along various dimensions, there is a need to support collection of disaggregated evidence to provide benchmarks for assessing the equity impacts of the various health programs.

### Limitations

The study is based on reported and documented perceptions of equity which may not necessarily translate into reality. Furthermore, while respondents talk about equity, the degree to which it is operationalized in their programming can only be examined through field research in the contexts where the programs are implemented. It was beyond the scope of the study to assess the actual equity impact of the DAP programs.

## Conclusions and recommendations

Our results demonstrate the difficulties in operationalizing equity and social justice in priority setting. It is essential that DAPs develop a greater understanding of the multifaceted ways in which equity (and inequity) can manifest itself. Inequities are often complex, and those who are likely to be on the receiving end of equity-based programs are often unable to advocate for themselves. Relatedly, DAPs must engage in dialogue with the multifaceted needs of the communities they are assisting; as this paper has shown, the concept of equity can mean different things to different people.

At the cross-organizational level, there should be an explicit set of criteria for ranking equity priorities, with specific attention to the intersectional nature of equity and vulnerability. Not only would this would allow equity to be given greater importance among PS criteria, but also provide nuance in the conceptualization of equity. Focused attention to multiple, intersecting, inter- and intragroup differences among vulnerable populations can make operationalizing equity more meaningful for a wider range of equity seeking groups. While equity may not be as easily operationalized as some of the other factors (e.g., evidence, cost-effectiveness), once practical and pragmatic criteria are taken into consideration, there should be some sort of weighting to allow equity to come to the fore. An internationally recognized, inter-sectoral framework for articulating and operationalizing equity-based project would facilitate the development of systems that could be used to collaboratively address multiple, intersecting inequities and dimensions of vulnerability simultaneously by reducing (or eliminating) the favourable treatment of some marginalized groups over others.

In a system where research and evidence are privileged, a stronger body of equity-supporting research could have significant benefits for those who are most in need of support. There’s need for research to systematically assess how equity is operationalized in program implementation and the equity impact of the DAP programs.

## Data Availability

The nature of qualitative research makes it impossible for us to avail the data without compromising the participants’ confidentiality.

## Abbreviations

DAP: Development Assistance partners
HIC: High income countries
LIC: Low income countries
MNCH: Maternal, Newborn and child health
NCD: Non- communicable disease
PS: Priority setting
